# Factors associated with hospitalization among older patients with mild traumatic injuries presenting to the emergency department in Korea: a retrospective observational study

**DOI:** 10.7586/jkbns.26.011

**Published:** 2026-05-26

**Authors:** Songhee Jeong, Younghui Hwang

**Affiliations:** 1 Emergency Department, Chungbuk National University Hospital, Cheongju, Korea; 2 College of Nursing and Research Institute of Nursing Science, Chungbuk National University, Cheongju, Korea

**Keywords:** Emergency room visits, Aged, Trauma nursing, Triage

## Abstract

**Purpose:**

This study aimed to identify factors associated with hospitalization among older adults with mild traumatic injuries who presented to a regional emergency medical center or regional trauma center.

**Methods:**

This retrospective secondary data analysis used medical records from trauma patients aged ≥ 65 years who were triaged as Korean Triage and Acuity Scale levels 4 or 5 and visited a regional emergency medical center or regional trauma center in Korea between January 1 and December 31, 2022. Of 38,371 visits during the study period, 442 older adults with mild traumatic injuries were included. Data on general characteristics, disease-related characteristics, medical treatments, and nursing interventions were analyzed using descriptive statistics, t-tests, *χ*² tests, and multivariate logistic regression analysis.

**Results:**

The 442 participants comprised 228 men (51.6%) and 214 women (48.4%); 225 participants (50.9%) were aged 65–74 years. In the multivariate analysis, longer emergency department stays were associated with a higher likelihood of hospitalization (odds ratio [OR] = 1.01; 95% confidence interval [CI], 1.00–1.01). Patients with fractures were more likely to be hospitalized (OR = 11.25; 95% CI, 1.12–111.78), whereas those who received counseling and education were less likely to be hospitalized (OR = 0.01; 95% CI, 0.00–0.04).

**Conclusion:**

These findings highlight the important role of emergency nursing care in the hospital admission process and support the need to incorporate nursing-related factors into future emergency department research and intervention strategies.

## INTRODUCTION

As life expectancy increases, population aging has intensified societal concerns related to the health, medical care, and welfare of older adults [1]. The growing prevalence of chronic diseases in this population has led to increased healthcare utilization, including greater use of emergency departments (EDs) [2,3]. According to the Emergency Medical Statistics Yearbook 2024, the number of individuals aged ≥ 60 years who visited emergency medical centers in Korea increased from 805,216 in 2010 to 2,393,081 in 2015, and further to 2,972,972 in 2024 [4].

ED patients in Korea are classified using the Korean Triage and Acuity Scale (KTAS) [5], a nationally standardized system that stratifies patients into five levels—resuscitation, emergent, urgent, less urgent, and non-urgent—based on clinical indicators such as vital signs, level of consciousness, and respiratory distress [5]. KTAS levels 1 and 2 represent life-threatening or potentially life-threatening conditions requiring immediate or rapid intervention. KTAS level 3 indicates cases in which the potential for progression to a condition requiring urgent treatment should be considered. In contrast, KTAS levels 4 and 5 refer to conditions that require treatment or reassessment within 1–2 hours or have a low likelihood of clinical deterioration [5]. Under the policy of the Ministry of Health and Welfare, KTAS levels 1–3 are designated as emergency cases, whereas levels 4–5 are categorized as non-emergency cases [6]. Among older adults visiting emergency medical centers, most were classified as KTAS level 3 (46.0%) or levels 4–5 (39.8%), accounting for 87.8% of visits, compared with 11.1% for KTAS levels 1 (2.7%) and 2 (8.4%) combined [7]. The high proportion of older adults presenting with low-acuity conditions poses challenges for emergency care delivery.

Older adults are particularly vulnerable to trauma because of age-related declines in sensory function and reflexes, as well as a higher prevalence of chronic comorbidities [8]. Reduced physiological reserve may limit compensation for minor injuries, increasing the risk of severe disability and trauma-related mortality [9]. In addition, geriatric conditions often present with atypical or nonspecific symptoms [10], leading to more extensive diagnostic testing and prolonged ED stays. It has been reported that hospitalization and prolonged ED stays of older patients is associated with a decline in activities of daily living (ADL) and an increased incidence of complications, including pneumonia, acute kidney injury, and urinary tract infections and mortality [11]. These factors may impose physical and psychological burdens on older adults. Greater attention is therefore needed to ensure that older adults with non-urgent conditions receive appropriate treatment and care in the ED, thereby reducing unnecessary hospitalization.

When older adults with minor injuries seek care at regional emergency or trauma centers—facilities primarily focused on critically ill patients—treatment delays may occur. In Korea, local emergency or trauma centers are designated to provide appropriate trauma care within each metropolitan city and province, including management of minor traumatic injuries [12]. Utilization of local centers may therefore facilitate more timely and appropriate care for older adults with minor trauma. However, restricting access to regional emergency centers without considering individual clinical characteristics may be inappropriate, as some patients with minor trauma require additional interventions, including hospitalization. Accordingly, analyzing the characteristics of older adults with minor trauma who present to regional emergency or trauma centers is necessary to inform guidelines that direct patients to appropriate facilities and enable them to receive timely care.

Patients requiring hospitalization may receive different types and intensities of nursing interventions than those discharged from the ED [13]. However, few studies have examined the nursing interventions provided to older adults with minor trauma who visit regional emergency medical centers, particularly the differences in nursing interventions between hospitalized and non-hospitalized patients. Additionally, few studies have investigated the nursing interventions provided to patients with minor trauma classified as KTAS levels 4 and 5, including conditions such as simple lacerations or minor fractures. A previous study [14] identified associations between hospitalization and general characteristics such as age, KTAS level, mode of arrival, and ambulance use among patients with mild traumatic injuries. Therefore, the purpose of this study was to examine differences in patient characteristics, nursing intervention, and medical treatment among older adults with minor trauma visiting the ED, comparing those admitted to the ward with those discharged from the ED. This study was conducted to identify factors associated with hospitalization among older adults with minor trauma presenting to the ED and to provide a basis for developing nursing intervention strategies for these patients.

## METHODS

### 1. Study design

This retrospective secondary analysis used medical record data to examine the characteristics of adults aged ≥ 65 years who visited the regional emergency medical center or regional trauma center of Chungbuk National University Hospital. This study is reported according to the Strengthening the Reporting of Observational Studies in Epidemiology (STROBE) statement.

### 2. Participants

**Figure 1.**
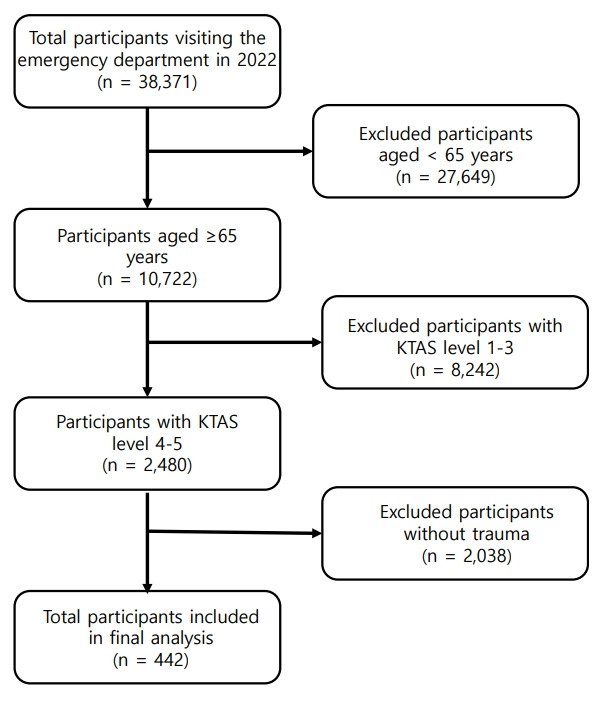
Flowchart of participant enrollment. KTAS = Korean Triage and Acuity Scale.

The study population comprised trauma patients aged ≥ 65 years with KTAS levels 4 or 5 who presented to the regional emergency medical center or regional trauma center of Chungbuk National University Hospital between January 1 and December 31, 2022. During this period, 38,371 visits were recorded, of which 10,722 involved adults aged ≥ 65 years. Among these, 2,480 were classified as mild conditions (KTAS levels 4 or 5), and 442 were trauma patients, constituting the final sample (Figure 1). Trauma patients were defined as individuals assigned injury-related codes under the 8th Korean Standard Classification of Diseases (KCD-8)—including S00–S99, T00–T35, T66–T75, T79–T88, and T90–T98 [15]—corresponding to injury of head (S00–S09), injury of neck (S10–S19), injury of thorax (S20–S29), injury of abdomen, lower back, lumbar spine and pelvis (S30–S39), injury of shoulder and upper arm (S40–S49), injury of elbow and forearm (S50–S59), injury of wrist and hand (S60–S69), injury of hip and thigh (S70–S79), injury of knee and lower leg (S80–S89), injury of ankle and foot (S90–S99), injuries involving multiple body regions (T00–T07), injuries to unspecified part of trunk, limb or body region (T08–T14), effects of foreign body entering through natural orifice (T15–T19), burns and corrosions of external body surface, specified by site (T20–T25), burns and corrosions confined to eye and internal organs (T26–T28), burns and corrosions of multiple and unspecified body regions (T29–T32), frostbite (T33–T35), other and unspecified effects of external causes (T66–T75), certain early complications of trauma (T79), complications of surgical and medical care, not elsewhere classified (T80–T88), sequelae of injuries, poisoning and other consequences of external causes (T90–T98). In the analysis of outcomes, injuries site based on the ICD classification were categorized, according to prior literature [16], into head or neck injury (S00–S19), thoracic injury (S20–S29), abdomen or pelvic injury (S30–S39), extremity injury (S40–S99), and others (T00–T35, T66–T75, T79–T88, and T90–T98). Patients presenting with trauma-related complaints, such as injury, falls, or lacerations, were also classified as trauma cases.

### 3. Instruments

#### 1) General characteristics

Sociodemographic variables included sex, age, type of health insurance (national health insurance, medical aid, special calculation provisions, others), and residence in a long-term care facility prior to the ED visit. Visit-related characteristics included visit day (weekday or weekend), arrival route (private vehicle or ambulance), visit time (daytime or nighttime), KTAS level, ED length of stay, and outcome (hospitalization or discharge home). Visit time was categorized as daytime (9:00 AM–6:00 PM) and nighttime (6:00 PM–9:00 AM the following day). KTAS is a five-level triage system used in South Korean EDs to prioritize care based on clinical urgency [5]. Adapted from the Canadian Triage and Acuity Scale, it classifies patients from Level 1 (resuscitation) to Level 5 (non-urgent) based on presenting symptoms, vital signs, and modifiers. Level 4 is defined as less urgent, requiring treatment or reassessment within 1–2 h, considering age, pain severity, and risk of deterioration. Level 5 is classified as non-urgent, referring to conditions that are not emergency in nature, are often related to chronic problems, or have a low likelihood of worsening. In this study, KTAS Levels 4 and 5 were defined as mild conditions.

#### 2) Disease-related characteristics and medical management

Disease-related characteristics included comorbidities, primary discharge diagnoses, and treatments provided during the ED visit. Comorbidities included hypertension, diabetes mellitus, cardiovascular disease, cerebrovascular disease, renal disease, and cancer. Diagnoses were coded according to the KCD-8 [17]. Medical management was categorized into eight groups based on Park et al. [18]: laboratory tests, medication, imaging studies, use of consumables, interventions, consultations, issuance of medical certificates, and physician consultation.

#### 3) Nursing intervention

Nursing interventions were classified into 12 domains based on Park et al. [19]: respiratory care, nutritional care, elimination care, exercise and posture maintenance care, comfort care, hygiene care, safety, spiritual support, counseling and education, medication administration communication, and patient and information management.

Respiratory care included assessment, airway maintenance, and oxygen therapy. Nutritional care involved assessment of nutritional status, nutritional support, and fluid and electrolyte management. Elimination care addressed defecation, urination, and drainage management. Exercise and posture maintenance care included assessment of ADL and range of motion (ROM), positioning and postural changes, and exercise-related care. Comfort care focused on physical and emotional comfort, and hygiene care addressed personal hygiene. Safety encompassed infection and accident prevention. Counseling and education included interviews and individual or group education. Communication involved coordination and referral to other healthcare professionals. Patient and information management included nursing handovers, implementation of physicians’ orders, and inspection and management of medical supplies and patient medications.

### 4. Data collection

This study employed a retrospective design and analyzed the medical record data of older adults aged 65 years or older with mild traumatic injuries who presented to the regional emergency medical center and the regional trauma center of Chungbuk National University in Chungcheongbuk-do over a one-year period, from January 1 to December 31, 2022. Data were collected by a researcher affiliated with Chungbuk National Hospital between November 1 and December 20, 2025, following approval from the Institutional Review Board (IRB) of Chungbuk National University.

### 5. Data analysis

Data analysis was conducted using SPSS version 27.0 (IBM Corp., Armonk, NY, USA) for Windows, and statistical significance was set at *p* < .050. Descriptive statistics summarized general characteristics, disease-related characteristics, medical management, and nursing interventions. Differences in hospitalization were examined using independent t-tests, *χ*2 tests, and Fisher’s exact test. Logistic regression analysis was performed to assess the associations between hospitalization (yes/no) and general characteristics, disease-related characteristics, medical management, and nursing interventions. Variables were selected using the enter method, and results were reported as odds ratios (ORs) with 95% confidence intervals (CIs).

### 6. Ethical considerations

This study was approved by the IRB of Chungbuk National University (Approval Number: CBNU-2025-A-0031).

## RESULTS

### 1. General characteristics of participants

A total of 442 participants were included, comprising 228 men (51.6%) and 214 women (48.4%). The largest age group was 65–74 years (n = 225, 50.9%). Most participants were covered by national health insurance, and more than two-thirds visited the ED by personal vehicle. At presentation, 438 patients (99.1%) were classified as KTAS level 4 and four patients (0.9%) as KTAS level 5. No changes in KTAS level were observed between ED admission and discharge (Table 1).

### 2. Differences in hospitalization according to general characteristics of participants

Significant differences were observed in hospitalization status according to general characteristics, including day of visit (*χ*² = 4.68, *p* = .030), time of visit (*χ*² = 4.72, *p* = .030), and length of ED stay (t = -12.31, *p* < .001). In contrast, no statistically significant differences in hospitalization were observed according to sex (*χ*² = 1.60, *p* = .206), age (*χ*² = 3.53, *p* = .171), type of insurance (*χ*² = 1.37, *p* = .714), or KTAS level (*χ*² = 0.34, *p* = 1.000) (Table 1).

### 3. Differences in hospitalization according to disease-related characteristics and medical treatment

Regarding treatment-related factors, hospitalization differed significantly according to the injury site (*χ*² = 44.45, *p* < .001), injury type (*χ*² = 53.04, *p* < .001), performance of laboratory tests (*χ*² = 47.95, *p* < .001), imaging studies (*χ*² = 11.81, *p* < .001), medication administration (*χ*² = 6.25, *p* = .011), use of consumables (*χ*² = 13.32, *p* < .001), and collaboration requests (*χ*² = 14.72, *p* < .001). In contrast, no statistically significant differences in hospitalization were observed according to disease-related characteristics (Table 2).

### 4. Differences in hospitalization according to nursing intervention

Regarding nursing interventions, hospitalization differed significantly according to the provision of elimination care (*χ*² = 38.40, *p* < .001), exercise and posture maintenance care (*χ*² = 29.24, *p* < .001), counseling and education (*χ*² = 29.95, *p* < .001), medication administration (*χ*² = 14.46, *p* < .001), and communication (*χ*² = 6.09, *p* =.014). In contrast, no statistically significant differences in hospitalization were observed according to respiratory care (*χ*² = 0.10, *p* = .732), comfort care (*χ*² = 0.54, *p* = .353), and hygiene care (*χ*² = 5.07, *p* =.148). Nutritional care and spiritual support were not provided to all patients. Safety and patient information management were provided to all patients (Table 3).

### 5. Factors associated with hospitalization

Logistic regression analysis was conducted to identify the factors associated with hospitalization. The independent variables included general characteristics that showed significant differences in hospitalization (day of visit, time of visit, and length of stay in the ED), disease or treatment-related factors (injury site, injury type, laboratory tests, imaging tests, medication administration, use of consumables, and collaboration), and nursing interventions (elimination care, exercise and posture maintenance care, counseling and education, medication administration, and communication) (Table 4).

In the univariate analyses, weekday visits (OR = 2.63; 95% CI, 1.06–6.49) and prolonged ED length of stay (OR = 1.20; 95% CI, 1.05–1.36) were associated with increased odds of hospitalization. Conversely, nighttime visits were associated with decreased odds of hospitalization (OR = 0.44; 95% CI, 0.20–0.94) compared to daytime visits. The odds of hospitalization were higher for thoracic injuries (OR = 8.65; 95% CI, 1.34–55.65), abdominal and pelvic injuries (OR = 6.62; 95% CI, 1.23–35.63), and extremity injuries (OR = 19.00; 95% CI, 4.25–84.88) than for head and neck injuries. Additionally, the odds of hospitalization were higher in patients with fractures (OR = 13.63; 95% CI, 5.75–32.32) compared to those with non-fracture injuries. The odds for hospitalization increased when laboratory tests (OR = 57.97; 95% CI, 7.85–428.22), imaging studies (OR = 14.58; 95% CI, 1.97–107.76), medication (OR = 3.59; 95% CI, 1.24–10.39), use of consumables (OR = 7.02; 95% CI, 2.11–23.33), and collaborative request (OR = 3.96; 95% CI, 1.88–8.36) were performed. The odds for hospitalization also increased when nursing interventions, including elimination care (OR = 11.88; 95% CI, 4.58–30.84), exercise and positioning maintenance care (OR = 6.14; 95% CI, 2.97–12.70), medication administration (OR = 6.22; 95% CI, 2.15–17.98), and communication (OR = 3.32; 95% CI, 1.51–7.28), were performed. In contrast, the odds for hospitalization decreased when nursing intervention of counseling and education (OR = 0.10; 95% CI, 0.04–0.27) was provided.

In the multivariate analyses, longer ED length of stay was associated with increased odds of hospitalization (OR = 1.01; 95% CI, 1.00–1.01). Fracture patients had higher odds of hospitalization (OR = 11.25; 95% CI, 1.12–111.78), whereas those who received counseling and education had lower odds (OR = 0.01; 95% CI, 0.00–0.04) (Table 4). The Hosmer–Lemeshow test indicated good model fit (*p* = .959). Nagelkerke’s R² was 0.81, indicating a high level of model explanatory power.

## DISCUSSION

This study identified factors associated with hospitalization among older adults with mild traumatic injuries admitted to the ED. In the multivariate analyses, hospitalization was significantly associated with ED length of stay, injury type, and nursing intervention of counseling and education. Among hospitalized patients, the ED length of stay was longer, and fractures were more common. In contrast, discharged patients were more likely to receive nursing consultation and education.

Most patients with minor trauma were discharged without hospitalization, and more than half presented to a regional trauma center or regional emergency medical center by private vehicle during daytime on weekdays. This finding is partly consistent with previous studies indicating that trauma incidents occur more frequently during the daytime, when activity levels are higher [20]. Regarding nursing interventions, medication administration was more frequently provided to hospitalized patients, whereas respiratory nursing was not associated with hospitalization in this study. This finding is partly consistent with previous study [21] reporting that medication was the most frequently performed nursing intervention before major trauma surgery, however, differs from study [22] that has identified respiratory nursing as a key competency for nurses in major trauma care. This may be explained by the relatively low likelihood of respiratory complications among older adults with minor trauma, resulting in less frequent provision of respiratory nursing. In contrast, patients who are hospitalized and require ongoing monitoring may be more likely to receive continued nursing interventions, such as medication.

Among older adults with mild traumatic injuries, ED length of stay was significantly associated with hospitalization, which is consistent with the findings of previous studies [23,24]. A prolonged stay in the ED generally reflects the need for more extensive medical management and nursing care and can therefore be considered a factor associated with hospitalization. However, prolonged time to hospitalization due to delays in treatment or decision-making in the ED may adversely affect patient prognosis [25-27]. In this study, the direct causes of prolonged ED length of stay were not recorded; therefore, the underlying reasons could not be determined. Future research is needed to examine whether the time from ED presentation to hospitalization is delayed among older adults with minor trauma, to identify the contributing factors, and to develop strategies to ensure timely and appropriate care from ED presentation to ward admission.

Fracture was significantly associated with hospitalization. It has been reported that fractures in older adults occur when bone fragility, such as osteoporosis, is combined with incidents such as falls [28]. Fractures in older adults can increase the risk of subsequent fractures, lead to disability, and increase mortality [29]. Therefore, even minor fracture injuries may warrant active management, including hospitalization. Accordingly, it is important to recognize that even minor fractures may require careful nursing care in older adults presenting to the ED. In addition, within the community, patients are screened for osteoporosis in advance to prevent fractures in older adults, and considerable emphasis is placed on rehabilitation that reduces bone fragility through nutritional supplementation, pharmacotherapy, and appropriate exercise [30]. A pathophysiological understanding of the relationship between osteopenia and fractures, as well as a pharmacological understanding of pharmacological treatment [31], is essential for rehabilitation nursing care of patients with fractures. Ongoing education is needed to ensure that ED nurses can provide appropriate nursing care based on biological nursing science knowledge related to fractures. Therefore, continued efforts are needed to identify strategies to reduce the incidence of fractures through the active management of osteoporosis among older adults in the community.

Nursing intervention of counseling and education were independently and significantly associated with hospitalization. Because counseling and education are generally more necessary for patients who are discharged than for those who are hospitalized [32,33], this finding may be explained by the greater provision of counseling and discharge education to patients discharged from the ED. Discharge education is particularly important for patients with trauma to provide information about self-management of their situation and prevent complications related to their injuries [32]. Given that a larger proportion of patients with mild trauma are discharged home rather than hospitalized, greater emphasis on counseling and education of discharged patients is warranted. Accordingly, the development of standardized discharge education materials for patients with mild traumatic injuries is recommended along with ongoing education and training for nurses in counseling and educational interventions. Further research is required to evaluate the effectiveness of these strategies.

The day and time of visit, which have been reported to influence outcomes among patients with mild traumatic injuries in a previous study [14], were not significantly associated with hospitalization in the multivariate analysis of this study. This finding likely reflects interactions with other covariates in the model. As these variables are closely related to regional characteristics and ED–specific factors [14], further investigations across diverse settings are warranted.

This study has a limitation in that it did not include all variables expected to be associated with hospitalization, such as injury severity and ADL. Because of the government-medical community conflict, the characteristics of patients hospitalized through the ED changed, making it impractical to use more recent data; therefore, data were collected in 2022, prior to the onset of this conflict. Because this study targeted patients who visited a regional trauma center or regional emergency medical center at a single hospital, there may be limitations in the generalizability of the findings. However, while most ED-related studies aimed at reducing overcrowding have focused primarily on critically ill patients, this study is meaningful because it examined patients with mild conditions. In particular, this study is significant in that it identifies differences in the application of nursing interventions between hospitalized and non-hospitalized patients. And the study highlights the importance of ED nurses’ biological nursing science knowledge and suggests directions for future education regarding nursing interventions performed in the ED.

## CONCLUSION

Importantly, this study is meaningful in that it demonstrates a strong association between nursing interventions and hospitalization among older adults with mild traumatic injuries. Older patients with minor trauma are more often discharged than hospitalized, highlighting the importance of counseling and discharge education for self-management and complication prevention. Accordingly, standardized discharge education materials and ongoing training for nurses are needed, along with further research to evaluate their effectiveness. Fractures are significantly associated with hospitalization in older adults, and even minor fractures may require active management and careful nursing care due to risks of disability, recurrence, and mortality. Therefore, osteoporosis management, rehabilitation, and ongoing education for ED nurses are essential to prevent fractures and provide appropriate, evidence-based care. The findings are significant because they provide an empirical foundation for continued and expanded research on ED nursing practices, particularly those that may influence clinical decision-making and patient disposition in trauma care.

## Figures and Tables

**Table 1.** Differences in Hospitalization According to General Characteristics of Participants (N = 442)

Variables	Admission (n = 34)	Discharge to home (n = 408)	Total	*χ*^2^ or t (*p*)
Sex				1.60 (.206)
Men	14 (41.2)	214 (52.5)	228 (51.6)	
Women	20 (58.8)	194 (47.5)	214 (48.4)	
Age (years)	76.74 ± 6.84	75.36 ± 7.71		3.53 (.171)
65–74	13 (38.2)	212 (52.0)	225 (50.9)	
75–84	17 (50.0)	139 (34.1)	156 (35.3)	
≥85	4 (11.8)	57 (14.0)	61 (13.8)	
Insurance type				1.37 (.714)
National health insurance	31 (91.3)	342 (83.8)	373 (84.4)	
Medical aid	1 (2.9)	18 (4.4)	19 (4.3)	
Special calculation provisions	1 (2.9)	19 (4.7)	20 (4.5)	
Others	1 (2.9)	29 (7.1)	30 (6.8)	
Residence in a long-term care facility prior to the ED				
No	34 (100.0)	408 (100.0)	442 (100)	
Visiting route				1.39 (.238)
Ambulance	12 (35.3)	106 (26.0)	118 (26.7)	
Personal vehicle	22 (64.7)	302 (74.0)	324 (73.3)	
Visit day				4.68 (.030)
Weekend	6 (17.6)	147 (36.0)	153 (34.6)	
Weekday	28 (82.4)	261 (64.0)	289 (65.4)	
Visit time				4.72 (.030)
Daytime (9:00–17:59)	24 (70.6)	209 (51.2)	233 (52.7)	
Nighttime (18:00–08:59)	10 (29.4)	199 (48.8)	209 (47.3)	
Length of stay in ED (minutes)	532.97 ± 285.56	190.38 ± 140.25		-12.31 (< .001)
Triage level at admission				0.34 (1.000)^†^
4	34 (100.0)	404 (99.0)	438 (99.1)	
5	0	4 (1.0)	4 (0.9)	
Triage level at discharge				0.34 (1.000)^†^
4	34 (100.0)	404 (99.0)	438 (99.1)	
5	0	4 (1.0)	4 (0.9)	

Values are presented as the mean ± standard deviation or n (%). ED = Emergency department.

† Fisher’s exact test.

**Table 2.** Differences in Hospitalization According to Disease-related Characteristics and Medical Management (N = 442)

Variables	Admission (n = 34)	Discharge to home (n = 408)	Total	*χ*^2^ or t (*p*)
Underlying disease				
Hypertension				0.20 (.659)
No	20 (58.8)	224 (54.9)	244 (55.2)	
Yes	14 (41.2)	184 (45.1)	198 (44.8)	
Diabetes				0.00 (.974)
No	26 (76.5)	311 (76.2)	337 (76.2)	
Yes	8 (23.5)	97 (23.8)	105 (23.8)	
Cardiovascular disease				0.02 (.890)
No	27 (79.4)	328 (80.4)	355 (80.3)	
Yes	7 (20.6)	80 (19.6)	87 (19.7)	
Cerebrovascular disease				0.41 (.630)
No	30 (88.2)	343 (84.1)	373 (84.4)	
Yes	4 (11.8)	65 (15.9)	69 (15.6)	
Renal disease				1.01 (.496)^†^
No	33 (97.1)	377 (92.4)	410 (92.8)	
Yes	1 (2.9)	31 (7.6)	32 (7.2)	
Cancer				0.19 (1.000)^†^
No	31 (91.2)	362 (88.7)	393 (88.9)	
Yes	3 (8.8)	46 (11.3)	49 (11.1)	
Injury site				44.45 (< .001)^†^
Head or neck injury	2 (5.9)	98 (24.0)	100 (22.6)	
Thoracic injury	3 (8.8)	17 (4.2)	20 (4.5)	
Abdomen or pelvic injury	5 (14.7)	37 (9.1)	42 (9.5)	
Extremity injury	19 (55.9)	49 (12.0)	68 (15.4)	
Others	5 (14.7)	207 (50.7)	212 (48.0)	
Injury type				53.04 (< .001)
Fracture	27 (79.4)	90 (22.1)	117 (26.5)	
Non-fracture	7 (20.6)	318 (77.9)	325 (73.5)	
Medical management				
Laboratory test				47.95 (< .001)
No	1 (2.9)	260 (63.7)	261 (59.0)	
Yes	33 (97.1)	148 (36.3)	181 (41.0)	
Imaging studies				11.81 (< .001)
No	1 (2.9)	125 (30.6)	126 (28.5)	
Yes	33 (97.1)	283 (69.4)	316 (71.5)	
Medication				6.25 (.011)
No	4 (11.8)	132 (32.4)	136 (30.8)	
Yes	30 (88.2)	276 (67.6)	306 (69.2)	
Use of consumables				13.32 (< .001)
No	3 (8.8)	165 (40.4)	168 (38.0)	
Yes	31 (91.2)	243 (59.6)	274 (62.0)	
Intervention				0.02 (.891)
No	17 (50.0)	199 (48.8)	216 (48.9)	
Yes	17 (50.0)	209 (51.2)	226 (51.1)	
Collaboration request				14.72 (< .001)
No	11 (32.4)	267 (65.4)	278 (62.9)	
Yes	23 (67.6)	141 (34.6)	164 (37.1)	
Issuance of a medical certificate				2.49 (.152)^†^
No	34 (100.0)	380 (93.1)	414 (93.7)	
Yes	0	28 (6.9)	28 (6.3)	
Physician consultation				0.42 (1.000)^†^
No	34 (100.0)	403 (98.8)	437 (98.9)	
Yes	0	5 (1.2)	5 (1.1)	

Values are presented as the mean ± standard deviation or n (%).

† Fisher’s exact test.

**Table 3.** Differences in Hospitalization According to Nursing Intervention (N = 442)

Variables	Admission (n = 34)	Discharge to home (n = 408)	Total	*χ*^2^ or t (*p*)
Respiratory care				0.10 (.732)^†^
No	31 (91.2)	378 (92.6)	409 (92.5)	
Yes	3 (8.8)	30 (7.4)	33 (7.5)	
Nutritional care				-
No	34 (100.0)	408 (100.0)	442 (100.0)	
Yes	0	0	0	
Elimination care				38.40 (< .001)^†^
No	25 (73.5)	396 (97.1)	421 (95.2)	
Yes	9 (26.5)	12 (2.9)	21 (4.8)	
Exercise and posture maintenance care				29.24 (< .001)
No	14 (41.2)	331 (81.1)	345 (78.1)	
Yes	20 (58.8)	77 (18.9)	97 (21.9)	
Comfort care				0.54 (.353)^†^
No	32 (94.1)	394 (96.6)	426 (96.4)	
Yes	2 (5.9)	14 (3.4)	16 (3.6)	
Hygiene care				5.07 (.148)^†^
No	33 (97.1)	407 (99.8)	440 (99.5)	
Yes	1 (2.9)	1 (0.2)	2 (0.5)	
Safety				-
No	0	0	0	
Yes	34 (100.0)	408 (100.0)	442 (100.0)	
Spiritual support				-
No	34 (100.0)	408 (100.0)	442 (100.0)	
Yes	0	0	0	
Counseling and education				29.95 (< .001)
No	29 (85.3)	152 (37.3)	181 (41.0)	
Yes	5 (14.7)	256 (62.7)	261 (59.0)	
Medication				14.46 (< .001)
No	4 (11.8)	185 (45.3)	189 (42.8)	
Yes	30 (88.2)	223 (54.7)	253 (57.2)	
Communication				6.09 (.014)
No	9 (26.5)	222 (54.4)	231 (52.3)	
Yes	25 (73.5)	186 (45.6)	211 (47.7)	
Patient and information management				0.08 (1.000)^†^
No	0	1 (0.2)	1 (0.2)	
Yes	34 (100.0)	407 (99.8)	441 (99.8)	

Values are presented as the mean ± standard deviation or n (%).

† Fisher’s exact test.

**Table 4.** Factors Associated with Hospitalization (N = 442)

Independent variables	Hospitalization			
	Univariate		Multivariate	
	OR (95% CI)	*p*	aOR (95% CI)	*p*
General characteristics				
Visit day (ref = weekend)	2.63 (1.06–6.49)	.036	1.43 (0.18–11.36)	.731
Visit time (ref = daytime)	0.44 (0.20–0.94)	.034	0.43 (0.09–2.16)	.308
Length of stay in the ED	1.20 (1.05–1.36)	.008	1.01 (1.00–1.01)	< .001
Injury site				
Thoracic injury (ref = head or neck injury)	8.65 (1.34–55.65)	.023	6.03 (0.15–246.41)	.342
Abdomen or pelvic injury (ref = head or neck injury)	6.62 (1.23–35.63)	.028	1.48 (0.09–23.53)	.781
Extremity injury (ref = head or neck injury)	19.00 (4.25–84.88)	< .001	0.73 (0.04–12.59)	.827
Others (ref = head or neck injury)	1.18 (0.23–6.21)	.842	0.50 (0.04–6.86)	.605
Injury type				
Fracture (ref = non-fracture)	13.63 (5.75–32.32)	< .001	11.25 (1.12–111.78)	.040
Medical interventions				
Laboratory test (ref = No)	57.97 (7.85–428.22)	< .001	8.26 (0.30–224.68)	.210
Imaging studies (ref = No)	14.58 (1.97–107.76)	.009	71.35 (0.82–6200.55)	.061
Medication (ref = No)	3.59 (1.24–10.39)	.019	0.20 (.002–16.92)	.473
Use of consumables (ref = No)	7.02 (2.11–23.33)	.001	4.05 (0.15–111.96)	.409
Collaboration request (ref = No)	3.96 (1.88–8.36)	< .001	3.52 (0.50–24.29)	.208
Nursing interventions				
Elimination care (ref = No)	11.88 (4.58–30.84)	< .001	3.33 (0.31–36.29)	.323
Exercise and posture maintenance care (ref = No)	6.14 (2.97–12.70)	< .001	1.43 (0.20–10.52)	.723
Counseling and education (ref = No)	0.10 (0.04–0.27)	< .001	0.01 (0.00–0.04)	< .001
Medication administration (ref = No)	6.22 (2.15–17.98)	< .001	0.69 (0.01–41.72)	.859
Communication (ref = No)	3.32 (1.51–7.28)	.003	1.23 (0.17–8.99)	.836

OR = Odds ratio; CI = Confidence interval; aOR = Adjusted odds ratio; Ref = Reference group; ED = Emergency department.

## References

[1] Gianfredi V, Nucci D, Pennisi F, Maggi S, Veronese N, Soysal P (2025). Aging, longevity, and healthy aging: the public health approach. Aging Clinical and Experimental Research.

[2] Günay Tuzcu G, Ekşi A (2025). Determinants of emergency medical services utilization among older adults: a comprehensive scoping review. Geriatric Nursing.

[3] Memedovich A, Asante B, Khan M, Eze N, Holroyd BR, Lang E (2024). Strategies for improving ED-related outcomes of older adults who seek care in emergency departments: a systematic review. International Journal of Emergency Medicine.

[4] Ministry of Health and Welfare (2025). 2024 annual report on emergency medical statistics [Internet]. https://www.mohw.go.kr/board.es?act=view&bid=0043&cg_code=&list_depth=1&list_no=1487814&mid=a10107010100&tag=&utm_source=chatgpt.com.

[5] Kim S, Kang H, Cho Y, Lee H, Lee SW, Jeong J (2021). Emergency department utilization and risk factors for mortality in older patients: an analysis of Korean National Emergency Department Information System data. Clinical and Experimental Emergency Medicine.

[6] Kim MJ, Kwon JH, Park SH, Byun YH, Song HY, Kim JH (2026). Nationwide age-specific changes in EMS-transported emergency department visits in Korea during the pre-COVID-19 and post-COVID-19 periods. Journal of Clinical Medicine.

[7] Park HA, Kim T, Kim HJ, Han SH (2025). Epidemiological trends in emergency department visits by age group: a report from the National Emergency Department Information System (NEDIS) of Korea, 2020-2024. Clinical and Experimental Emergency Medicine.

[8] Francesco V, Roberto B, Giulia C, Piero CS, Michele A, Andrea S (2022). All elderly are fragile, but some are more fragile than others: an epidemiological study from one of the busiest trauma centers in Italy. Updates in Surgery.

[9] Tu HR, Lin TH, Hsu LM, Wu IH (2025). High mortality and medical costs in geriatric trauma patients: surgical treatment and risk factors from a retrospective cohort study at a Level I trauma center. Annals of Geriatric Medicine and Research.

[10] Van Dam CS, Peters MJL, Hoogendijk EO, Nanayakkara PWB, Muller M, Trappenburg MC (2023). Older patients with nonspecific complaints at the Emergency Department are at risk of adverse health outcomes. European Journal of Internal Medicine.

[11] Bannelier H, Zerah L, Catoire P, Phagouapé J, Guyot S, Freund Y (2025). Association between time to geriatric ward admission and change in functional status in older adults after an emergency department visit: a prospective cohort study. Internal and Emergency Medicine.

[12] Ministry of Government Legislation (2026). Emergency Medical Services Act [Internet]. https://www.law.go.kr/lsSc.do?section=&menuId=1&subMenuId=15&tabMenuId=81&eventGubun=060101&query=%EC%9D%91%EA%B8%89%EC%9D%98%EB%A3%8C%EC%97%90+%EA%B4%80%ED%95%9C+%EB%B2%95%EB%A5%A0#undefined.

[13] Tacbas M, McGovern B, Rodricks J (2023). Closing the gap: the role of discharge nurses in an emergency department. Journal of Emergency Nursing.

[14] Oh MT, Lee SH, Park SW, Park SC, Kim HB, Jo YM (2018). Factors associated hospital admission in patients with low acuity visiting emergency department. Journal of the Korean Society of Emergency Medicine.

[15] Hwang YS, Jo HY, Yoo HW, Kim YM, Kim HY (2020). Characteristics of children with trauma compared to those with disease in the emergency department: a Korean single regional emergency medical center study. Pediatric Emergency Medicine Journal.

[16] Cho OH, Yoon J (2023). Characteristics and clinical outcomes of older patients with trauma visiting the emergency department before and during the COVID-19 pandemic: a level 1 trauma center cohort study. Journal of Korean Gerontological Nursing.

[17] Korea Disease Control and Prevention Agency (2021). Korean Standard Classification of Diseases and Causes of Death (8th rev.) [Internet]. https://kcdcode.kr/browse/contents/0#.

[18] Park SH, Jang YS, Kim SJ (2019). Analysis of the current state of home health nursing for elderly patients in advanced general hospital. Journal of Korean Clinical Nursing Research.

[19] Park JH, Sung YH, Song MS, Cho JS, Sim WH (2000). The classification of standard nursing activities in Korea. Journal of Korean Academy of Nursing.

[20] Lee HR (2020). Relationship between injury mechanism, trauma site and hospitalization of elderly trauma patients visited through emergency medical center [master’s thesis].

[21] Yang SK (2022). Analysis of nursing intervention for severe trauma patients before operated in trauma center [master’s thesis].

[22] Kim YH (2024). Development of nursing competency and behavioral indicators of major trauma in the resuscitation zone of regional trauma centers [dissertation].

[23] van Dijk M, Gaakeer MI, Jonker M, Baden DN, de Groot B (2026). The association between emergency department length of stay and hospital length of stay: an observational multi-centre cohort study. Internal and Emergency Medicine.

[24] Curiati PK, Gil-Junior LA, Morinaga CV, Ganem F, Curiati JAE, Avelino-Silva TJ (2020). Predicting hospital admission and prolonged length of stay in older adults in the emergency department: the PRO-AGE scoring system. Annals of Emergency Medicine.

[25] García-Peña C, Pérez-Zepeda MU, Robles-Jiménez LV, Sánchez-García S, Ramírez-Aldana R, Tella-Vega P (2018). Mortality and associated risk factors for older adults admitted to the emergency department: a hospital cohort. BMC Geriatrics.

[26] Kim JY, Kim OH (2025). Recent advances in prehospital and in-hospital management of patients with severe trauma. Journal of Clinical Medicine.

[27] Li A, Feng Q, Zhao Y, Zhang X, Jiang W (2025). Comprehensive meta-analysis of emergency trauma outcomes: trends, interventions, and survival rates. Frontiers in Public Health.

[28] Yang ZC, Lin H, Jiang GH, Chu YH, Gao JH, Tong ZJ (2023). Frailty is a risk factor for falls in the older adults: a systematic review and meta-analysis. The Journal of Nutrition, Health and Aging.

[29] LeBoff MS, Greenspan SL, Insogna KL, Lewiecki EM, Saag KG, Singer AJ (2022). The clinician's guide to prevention and treatment of osteoporosis. Osteoporosis International.

[30] Das S, Kotwal V, Thakur A, T L, Kumar N (2025). Bone health and osteoporosis management in the elderly: a review of guidelines and orthopedic implications. Cureus.

[31] Snodgrass P, Zou A, Gruntmanis U, Gitajn IL (2022). Osteoporosis diagnosis, management, and referral practice after fragility fractures. Current Osteoporosis Reports.

[32] Carey A, Starkweather A, Bai A, Horgas A, Cho H, Beneciuk JM (2024). Emergency department discharge teaching interventions: a scoping review. Journal of Emergency Nursing.

[33] Moradi Rekabdar Kalaiee Z, Ghafouri R, Zandi M, Nasiri M (2024). Effect of implementing of the IDEAL discharge model on satisfaction of patient referred to trauma emergency department. PLoS One.

